# Probiotics in miRNA-Mediated Regulation of Intestinal Immune Homeostasis in Pigs: A Physiological Narrative

**DOI:** 10.3390/microorganisms12081606

**Published:** 2024-08-07

**Authors:** Valeria Bárcenas-Preciado, Verónica Mata-Haro

**Affiliations:** Laboratorio de Microbiología e Inmunología, Centro de Investigación en Alimentación y Desarrollo, AC (CIAD) Carretera Gustavo E. Astiazarán 46, Col. La Victoria, Hermosillo 83304, Mexico; vbarcenas221@estudiantes.ciad.mx

**Keywords:** probiotics, signaling pathways, regulation, microbiota, microRNA

## Abstract

The microbiota plays a crucial role in maintaining the host’s intestinal homeostasis, influencing numerous physiological functions. Various factors, including diet, stress, and antibiotic use, can lead to such imbalances. Probiotics have been shown to restore the microbiota, contributing to maintaining this balance. For instance, the weaning stage in piglets is crucial; this transition can cause unfavorable changes that may contribute to the onset of diarrhea. Probiotic supplementation has increased due to its benefits. However, its mechanism of action is still controversial; one involves the regulation of intestinal immunity. When recognized by immune system cells through membrane receptors, probiotics activate intracellular signaling pathways that lead to changes in gene expression, resulting in an anti-inflammatory response. This complex regulatory system involves transcriptional and post-transcriptional mechanisms, including the modulation of various molecules, emphasizing microRNAs. They have emerged as important regulators of innate and adaptive immune responses. Analyzing these mechanisms can enhance our understanding of probiotic–host microbiota interactions, providing insights into their molecular functions. This knowledge can be applied not only in the swine industry, but also in studying microbiota-related disorders. Moreover, these studies serve as animal models, helping to understand better conditions such as inflammatory bowel disease and other related disorders.

## 1. Introduction

A healthy gastrointestinal tract is populated by many microorganisms, forming the intestinal microbiota, which interacts mutually beneficially with the host. This interaction is closely connected to the regulation of immune responses. The microbiota is crucial for developing the immune system and its capacity to distinguish between commensal and pathogenic bacteria. It also plays a significant role in fostering tolerance and immunity to both own and foreign antigens. Thus, host–microbiota interactions are important to maintain homeostasis, including controlling inflammatory responses. These interactions occur in the different defense barriers and help maintain balance in the body. When the microbiota is deregulated, it can contribute to the loss of tolerance and develop inflammation and pathologies mediated by the immune system [1,2].

The gastrointestinal tract is continually exposed to diverse substances, such as dietary constituents and pathogenic microorganisms, capable of inducing alterations and activating defense mechanisms. One of the defense barriers is the mucous membranes, which cover much of the intestinal wall. The mucosa confers protection, secretion, and absorption of nutrients and immune functions. Disruptions in the organism’s equilibrium, exemplified by the evasion of mucosal defenses by pathogens, cause an inflammatory response, generating a sequence of cellular events that culminate in the production of proinflammatory mediators [3,4].

In the swine industry, the weaning stage is paramount, as the transition to solid food can damage the intestinal barrier, atrophy of intestinal villi, and overproduction of inflammatory cytokines. This elevates the likelihood of experiencing diarrhea due to dehydration or infection and reduced food intake, consequently impacting growth. Antibiotics have traditionally been employed to alleviate diarrhea and as growth promoters. However, due to pathogens’ escalating resistance to intestinal antibiotics and the adverse effects of drug residues, antibiotics have been banned in numerous countries. Currently, probiotics have been proposed as alternatives to antibiotics in weaned piglets [5,6,7]. For example, the multispecies probiotics with *Lactobacillus acidophilus*, *Lacticaseibacillus casei*, *Bifidobacterium thermophilum*, and *Enterococcus faecium* were able to reduce diarrhea caused by *Escherichia coli* F18 in weaned piglets, as well as increase villi height in the small intestine [8].

Probiotics, which are live microorganisms conferring benefits to the host, demonstrate anti-inflammatory effects, modulate the microbiota, enhance the functionality of the intestinal epithelial barrier, and alleviate diarrhea [9]. Bifidobacteria and lactic acid strains are among the most prevalent probiotics in certain foods and supplements [10]. Probiotic microorganisms are recognized by immune system cells through membrane receptors, notably TLR2, which recognizes peptidoglycan (PGN) [11,12]. Stimulation of these receptors activates key proteins within the signaling pathway, ultimately triggering transcription factors that induce the production of cytokines, such as interleukin-10 (IL-10). This cytokine is known for its anti-inflammatory action and is pivotal in maintaining intestinal homeostasis [9].

To better understand how probiotics work, delving deeper into their interaction with the host is necessary. First, how do cells recognize probiotics? Cells involved in innate immunity, such as macrophages, dendritic cells (DCs), and intestinal epithelial cells (IECs), can initiate immune responses by recognizing molecular structures, including microorganism-associated molecular patterns (MAMPs) through pattern recognition receptors (PRRs). Within these receptors are the Toll-like receptors (TLR). TLRs detect molecules of bacterial, viral, and fungal origin. When TLRs recognize a ligand, they initiate an intracellular signaling cascade where response proteins are activated or blocked, activating transcription factors that change the expression of several immune response genes, releasing molecular mediators such as interferons and cytokines [12,13].

Thus, the orchestrated response activates various signaling pathways, notably nuclear factor erythroid 2 p45-related factor 2 (Nrf-2), nuclear factor-kappa B (NF-κB), and mitogen-activated protein kinase (MAPK). Activation of these pathways prompts alterations in cellular gene expression, inducing the synthesis of effector molecules essential for immune defense mechanisms, encompassing both inflammatory and anti-inflammatory processes [12,14,15]. However, other molecules are involved in this process, such as microRNAs (miRNAs) which are short sequences of non-coding RNA (ncRNA). These miRNAs also play a crucial role in orchestrating immune responses by regulating gene expression at the post-transcriptional level. They help fine-tune the immune response, ensuring a balanced and effective defense mechanism. Additionally, probiotics can modulate the expression of miRNAs. For instance, when the function of miR-671-5p is blocked in porcine moDCs stimulated with *Bifidobacterium animalis* subsp. *lactis*, the expression of IL-10 increases. This suggests that miRNAs may act as modulators of molecular mediators [16].

However, the regulatory mechanisms are extensive and include the recognition of microbial structures, the activation of signaling pathways, the expression of response proteins, and the activation of transcription factors with the interaction of ncRNAs that function as regulatory keys [17,18]. This review aims to explain the recognition of probiotics in the intestine and their potential molecular regulation mechanisms. We will discuss how probiotics can influence the expression of miRNAs and how this interaction affects signaling pathways and overall health. Moreover, we will provide evidence of their use in farm animals, particularly pigs. Attempting to explain these interactions can help understand the mechanisms of action of probiotics. This narrative encompasses their journey from reaching the intestine to the potential changes that can occur in cells to orchestrate immune responses.

## 2. Intestinal Mucosal Immune System: Probiotics Pass through the Gastrointestinal Tract

The intestinal epithelium forms the inner lining of the small and large intestines, acting as the interface with the external environment. It maintains a controlled homeostatic system for molecular transport, balancing the various functions of the intestinal tract, including digestion, immunity, and tolerance, and supporting the diverse symbiotic roles of the microbiota [19]. Probiotics interact with intestinal epithelial and immune cells in various regions of the intestine, each offering specific conditions for different types of interactions. 

**Figure 1:**
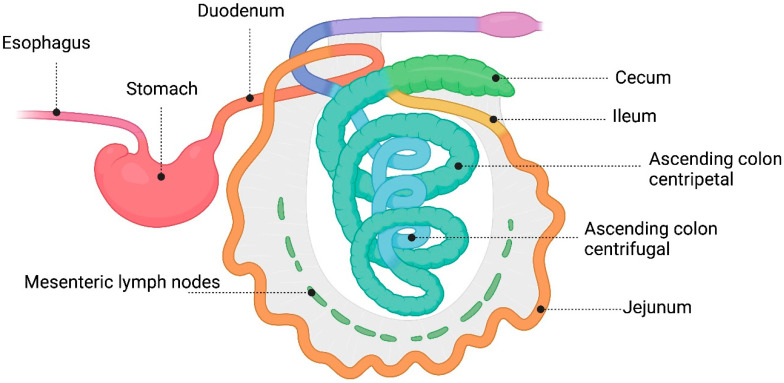
Gastrointestinal tract of pigs.

The small intestine is where digestion and absorption of micro- and macronutrients occur. Figure 1 shows the distribution of the small and large intestines of pigs. The small intestine comprises three sections: duodenum, jejunum, and ileum. The duodenum, the first part of the small intestine, receives a mixture of gastric contents with bile and pancreatic juices. Although the pH is less acidic than in the stomach, the presence of digestive enzymes can affect the viability of probiotics [20,21]. The jejunum in pigs comprises 80% of the small intestine and has villi that facilitate the absorption of nutrients broken down by enzymes in the duodenum. Vitamin B12 is absorbed in the ileum, and bile salts and other nutrients are reabsorbed [21]. Typically, the duodenum and jejunum have bacterial concentrations ranging from 10^3^ to 10^4^ bacteria/mL, which then rise to 10^8^ bacteria/mL in the ileum; this increase is due to the more basic pH in the ileum, creating a favorable environment for bacteria to break down and ferment carbohydrates and utilize energy [22]. 

The large intestine, the final part of the digestive system, is a long tube that includes the pig’s cecum, colon, and rectum. It absorbs fluids and electrolytes and acts as a barrier against microbial invasion. The health of the large intestine relies on a well-balanced microbiota [21]. The colon features an inner viscous layer and an outer less viscous layer, whereas the small intestine is characterized by a single mucosal layer [19].

**Figure 2:**
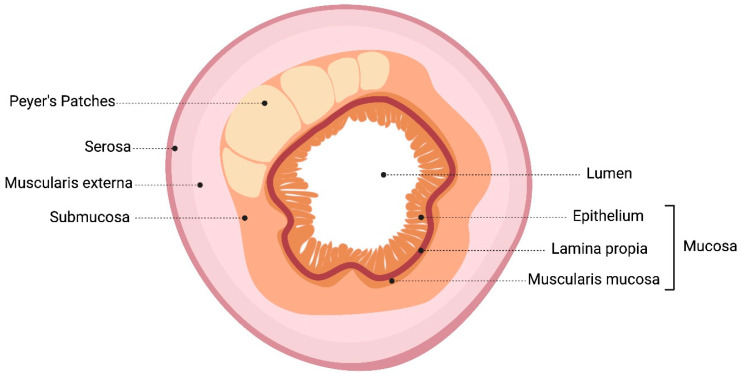
Schematic representation of the small intestine depicting the inner layers of the wall.

The different layers of the small intestine are depicted in Figure 2. The outermost layer of the intestine, the serosa, provides protection and minimizes friction. The muscularis externa, with its smooth muscle layers, drives peristaltic movements. The innermost layer, the mucosa, is critical for nutrient absorption, containing cells and glands that secrete digestive enzymes. Beneath the mucosa, the submucosa provides structural support and houses blood vessels and nerves [23]. The lumen within the mucosa is where alimentary content flows, allowing nutrient absorption and digestion. Together, these layers facilitate digestion and nutrient absorption and maintain the functional integrity of the gastrointestinal tract.

Intestinal epithelial cells (IECs) serve as the primary barrier against antigens in the intestinal lumen. This layer comprises various cell types, including enterocytes, Goblet cells, Paneth cells, tuft cells, neuroendocrine cells, and Microfold cells (M cells). Maintaining the health of the IEC layer is crucial for effective digestion, nutrient absorption, barrier integrity, and microbiota balance. The intestinal mucosal immune system is organized at induction and effector sites. The induction site mainly comprises gut-associated lymphoid tissue (GALT), which includes isolated lymphoid follicles (ILFs), Peyer’s patches (PPs), and mesenteric lymph nodes (MLNs). In these areas, immune cells such as M cells and antigen-presenting cells (APCs), including dendritic cells (DCs), macrophages, and IECs, play a vital role in antigen uptake and presentation to immune effector cells. The PPs are located throughout the ileum and jejunum segments, and are thus categorized as ileal PPs (IPPs) or jejunal PPs (JPPs). These are covered by follicle-associated epithelium-containing M cells. The effector sites comprise lymphocytes located both in the epithelial layer and in the lamina propria and encompass intestinal mucosal intraepithelial lymphocytes (IELs) and lamina propria lymphocytes (LPLs). The presentation of antigens by APCs to lymphocytes triggers the production of cytokines and antibodies, which are crucial for the immune response [23,24,25].

The immune response in the intestinal mucosa is initiated through antigen uptake from the induction site, with PPs being a key site for this induction. Antigen-transporting cells, specifically M cells, located in PPs, can phagocytize microorganisms and antigens. These antigens are then processed and presented to antigen receptor molecules and, ultimately, to helper T cells (Th). Upon activation, helper T cells secrete various cytokines that stimulate the differentiation and proliferation of B cells. These B cells produce significant amounts of secretory IgA (sIgA), which is released into the intestinal lumen and plays a vital role in enhancing the immune properties of the gut [24,26]. Thus, GALT is a crucial mediator of immune responses since it also induces the production of type I interferons (IFN-Is) by macrophages, DCs, and IECS. It is vital in orchestrating the body’s defense mechanisms against pathogens [27].

## 3. Probiotics Interact with Host Cell

Understanding the regulatory mechanisms of probiotics involves understanding how these microorganisms interact with host cells at the molecular level and how these interactions can influence health. First, there is an interaction with the microbiota; they also compete with pathogens for nutrients and space, reducing luminal pH, inhibiting microbial adherence, and producing antimicrobial substances that inhibit the growth of unwanted microorganisms [28,29]. Additionally, probiotics produce bioactive metabolites such as short-chain fatty acids (SCFAs), among others, that increase their mode of action. These are so-called postbiotics [30]. Probiotics of the genus *Bifidobacterium* and *Lactobacillus* can ferment dietary fibers to produce SCFAs such as butyrate and propionate, which have anti-inflammatory effects and can strengthen the intestinal barrier [31,32]. In a study by Oh J et al. (2021), piglets were supplemented with a multispecies probiotic blend (*Bacillus amyloliquefaciens*, *Limosilactobacillus reuteri*, and *Levilactobacillus brevis*). The results showed increased production of certain SCFAs, specifically acetate and formate, in the feces. They also investigated whether there was a correlation between these SCFAs and the microbiota. They found an association between *Olsenella* and *Lactobacillus* with acetate, and *Acidaminococcus* was significantly correlated with formate [33]. This suggests that probiotic supplementation can modulate the microbiota, in part, through the production of SCFAs. Furthermore, *Bifidobacterium* and *Lactobacillus* also produce vitamins (such as vitamin K and some B complexes) [34]. 

**Figure 3:**
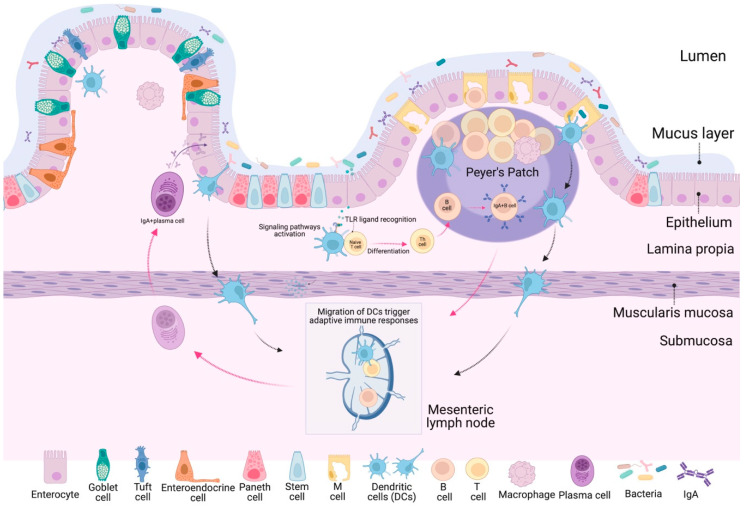
Interaction of immune cells in the small intestine. The small intestine modulates the immune system, integrating innate and adaptive immune responses. Innate immunity is rapidly activated in response to microorganisms, involving cells such as macrophages, dendritic cells, and M cells that capture and present antigens. Adaptive immune responses can originate in Peyer’s patches or mesenteric lymph nodes (MLNs). Bacteria are primarily sampled by DCs in Peyer’s patches after transcytosis through the specialized epithelium lining these lymphoid organs. Effector T lymphocytes and antibody-producing B cells reside within the intestinal lumen, particularly those producing Immunoglobulin A (IgA), which is essential in protecting mucosal surfaces by neutralizing infectious microorganisms and toxins. Furthermore, CD103+ DCs can migrate to MLNs and trigger adaptive immune responses. Together, these coordinated processes ensure an effective and balanced immune response, protecting the body from infections while maintaining intestinal homeostasis.

The immune system primarily comes into play in the mucosal layer of the gastrointestinal tract. First, ingested probiotics are recognized by IECs in the mucosal layer, where probiotics trigger innate responses and can modulate adaptive immune responses. In innate responses, DCs, and macrophages can directly recognize the microorganism and secrete a profile of cytokines [35]. The interaction of intestinal and immune cells with probiotics is shown in Figure 3. Chronic inflammation is characterized by an overexpression of inflammatory molecules for a prolonged period, and the intake of probiotics helps counteract this overproduction. When the levels of inflammatory cytokines decrease, the body returns to homeostasis. For this reason, probiotics are an excellent option to treat intestinal diseases.

The modulation of the immune response by probiotics encompasses intricate molecular and cellular mechanisms. Notably, stimulating innate and adaptive immune cells triggers phagocytosis and IgA secretion while concurrently amplifying Th1 responses and tempering Th2 responses [36]. Certain probiotics exhibit the capacity to induce the secretion of messenger proteins by macrophages and DCs. For instance, the production of IL-10 plays a pivotal role in regulating inflammatory diseases; however, specific probiotic recognition is a determining factor in the nature of the response [37]. That is, probiotics act through various molecular mechanisms; Table 1 shows some of their effects in different animal models. The results should be taken with caution since some of the results were obtained ex vivo with cells stimulated with probiotics.

Moreover, there is a synergistic interaction between the intestinal epithelium and the immune system, including the microbiota, which is crucial for proper gastrointestinal function. The direct interaction between these bacteria and the gastrointestinal epithelium has systemic effects due to its cellular impact. In vitro studies have demonstrated that bacteria with colonizing potential in the gastrointestinal system can stimulate intestinal cell lines, resulting in a certain cytokine profile involvement [38]. Probiotics can help maintain the intestinal barrier, reducing permeability and preventing the translocation of pathogens and toxins. The mechanism of action involves the production of proteins that strengthen the tight junctions between epithelial cells [39].

**Table 1:** Probiotics molecular activity in different animal models.

Probiotic	Effect	Model	Reference
*B. coagulans*	Increase level gene expression of *IFN-α*, *IFN-γ*, *OAS1*, *MX2*, *IL-4*, *CCL-2* in ileum	Pig	[40]
*B. animalis* subsp. *lactis*	Induces IL-10 production in moDCs	Pig	[41]
*B. animalis* subsp. *lactis*	Reduces TNF-α production	Mice	[42]
*B. breve*	Induces CD4+ T cells in colon	Mice	[28]
*B. subtilis*	Increase IgA and IgM in serum	Pig	[43]
*L. sobrius*	Increase IgA in serum	Pig	[44]
*L. acidophilus* W55, *B. lactis* W51	Induces FOXP3^+^ T reg, induces production of IL-10 in PBMCs	Human	[45]
*L. rhamnosus* GG, *L. casei* IMAU60214, *L. helveticus* IMAU70129	Induces NF-κB p65 in macrophages	Human	[46]

## 4. Probiotics Activate Signaling Pathways

The immune system cells have a series of receptors on their surface that recognize structures and thus trigger an immune response, activating intracellular signaling pathways. Among them are TLRs, which belong to the pattern-recognizing receptor (PRR) category and play a vital role in the innate immune system. They recognize microorganism-associated molecular patterns (MAMPs). When TLRs are activated, a signaling cascade is triggered that changes the gene expression of cells to produce cytokines and other mediators and thus regulate the immune response [47,48]. TLR2 primarily identifies peptidoglycan, a significant constituent of probiotic bacteria [49]; as a result, it is part of the complex interplay between the immune system and probiotic-induced effects.

The detection by the receptors of a “signal”, that is, the binding of ligands to TLR, initiates signaling pathways leading to the activation of specific intracellular proteins ending with changes in gene expression. The TLR-initiated pathway includes two distinct pathways: the MyD88 (myeloid differentiation factor 88)-dependent pathway is used by all TLRs (except TLR3) and leads to the production of inflammatory cytokines. The other is TRIF-dependent (adaptor-inducing interferon-β containing the TIR domain), used by TLR3 and TLR4, and is associated with the production of interferon type 1 (IFN-1) [47].

**Figure 4:**
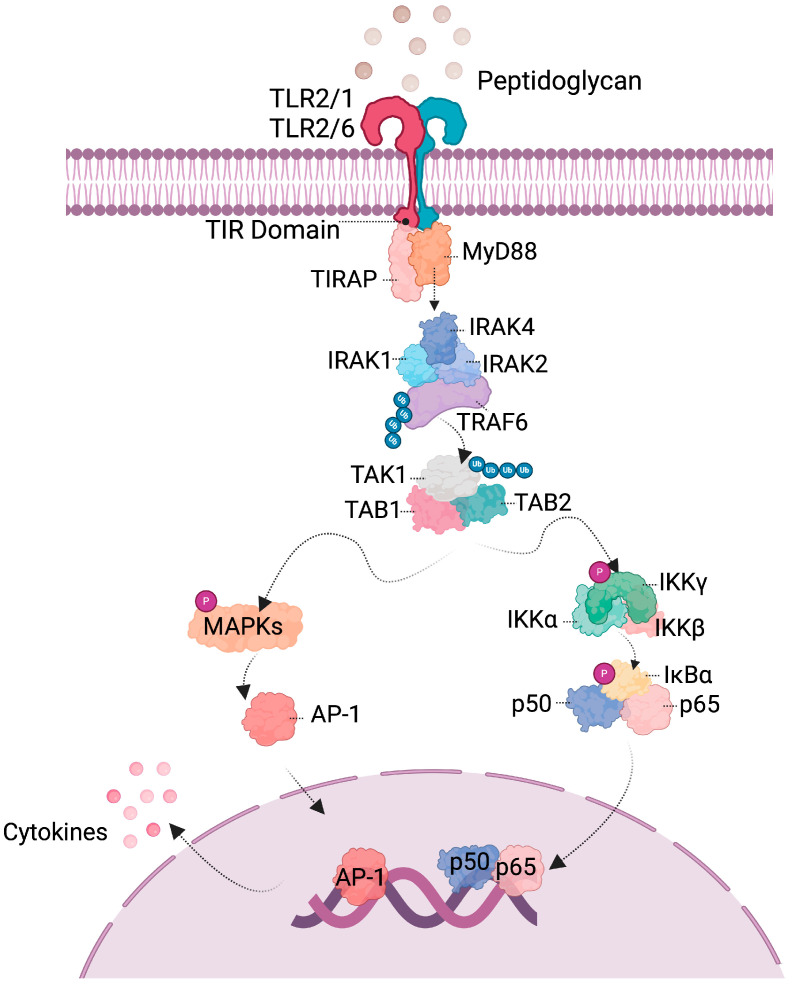
Signaling pathway involved in probiotics recognition. When bacterial structures are recognized by TLRs, a signaling cascade is activated. The recruitment of adaptor molecules such as MyD88 and TRAF6 leads to the activation of transcription factors belonging to MAPKs or NF-κB, resulting in changes in gene expression, including the production of molecular mediators such as cytokines.

Following recognition by TLR2, the signaling cascade originates from the intracellular domain of TIR (Toll/IL-1 receptor), comprising three binding sites for adapter proteins MyD88 and TIRAP (adaptor protein containing TIR domain). Subsequently, the involvement of TRAF6 (TNF receptor-associated factor), another adapter protein, directs the activation of MAPK and NF-κB. The ensuing step involves the translocation of transcription factors like AP1 (Activating Protein 1) or the p50 and p65 subunits to the nucleus to convey signals for alterations in gene expression. These alterations may involve proteins or other molecular mediators like cytokines within the cascade. Depending on the specific stimulus, the response can be either inflammatory or anti-inflammatory [47,50]. TLR activation triggers an immune response executed by modifying the expression of transcription factors and producing inflammatory mediators. A simplified representation of the signaling pathways involved is depicted in Figure 4.

## 5. microRNAs as Regulators of the Intestinal Gut Microbiota

Non-coding RNAs are crucial regulatory molecules in various cellular processes. They are classified by length into small ncRNA (sncRNAs) and long ncRNA (lncRNAs), functionality (regulatory and housekeeping), and location (cellular and exosomal). The microbiota can regulate coding and ncRNAs, with evidence showing microbiota-specific regulation of the intestinal host transcriptome in adult germ-free mice. Among sncRNAs, miRNAs are the most studied, regulating gene expression by binding to the target mRNA 3′ untranslated regions (3′ UTRs) [51].

**Figure 5:**
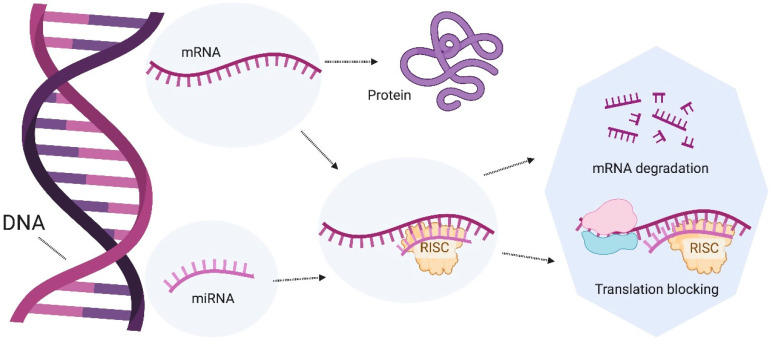
Mechanism of action of miRNAs. miRNAs bind to complementary sequences on mRNA and direct their degradation or inhibit their translation, thus modulating the protein produced from a specific gene. Abbreviations: microRNA, miRNA; messenger RNA, mRNA; RISC, RNA-induced silencing complex.

miRNAs are ncRNA molecules, generally 20–22 nucleotides in length, that play a crucial role in gene expression regulation. miRNAs bind to complementary sequences on messenger RNAs (mRNAs) and direct their degradation or inhibit their translation, thus modulating the protein produced from a specific gene (Figure 5). This post-transcriptional regulatory mechanism is fundamental for numerous biological processes, including development, cell differentiation, apoptosis, the cell cycle, and responses to immune challenges and stress. Its dysfunction is associated with multiple diseases, including cancer, cardiovascular diseases, neurological disorders, and inflammatory diseases [51]. Imbalances in the gut microbiota, primarily driven by gene expression patterns, are linked to gastrointestinal diseases and to conditions such as cancer, diabetes, cardiovascular diseases, obesity, and neurological developmental disorders [52].

The interaction of microbiota and the intestine’s immune cells involves numerous molecules, where certain types of RNA are key physiological regulators critical in health and disease states. When probiotics stimulate intestinal cells, miRNAs are also expressed, which have essential modulatory functions, including exerting anti-inflammatory effects on host cells. Therefore, miRNAs also play an essential role in modulating immune responses and maintaining intestinal homeostasis. 

Research on the role of miRNAs in gut health is in its early stages, but the results are promising for humans [53]; however, less is known for animal health. For example, recent studies have identified several miRNAs differentially expressed in healthy piglets’ intestines versus those with gut problems. These miRNAs could serve as biomarkers for gut health or as targets for therapeutic interventions since the expression of microRNAs changes when there is an alteration, for instance, when inflammation is induced in in vitro and in vivo models [54]. Rodríguez-Nogales et al. (2018) studied a DSS-colitis induction model in mice, where the expression of miR-155 and miR-223 were upregulated, while miR-143, miR-150, and miR-375 were downregulated, indicating that miRNAs are important post-transcriptional regulators [55].

## 6. Role of microRNAs in the Intestine

miRNAs are essential regulators of gene expression and are closely linked to the microbial community. The interactions between the host and the microbiota foster a healthy symbiotic relationship. However, an imbalance occurs when there is an unfavorable change in the microbial community, disrupting host–microbe interactions and potentially leading to various diseases. Hence, maintaining a balanced microbiota is essential for overall health [56].

Tian et al. (2019) observed an increase in the expression of miR-31 in the inflamed mucosa in patients with IBD (inflammatory bowel disease) and mice with DSS-induced colitis, where the NF-κB and STAT3 signaling pathways are involved [41]. One of the functions of this miRNA is to relieve inflammation and promote regeneration of epithelial cells [41]. In another study, Xue et al. (2011) observed that commensal bacteria downregulated dendritic cell miR-10a expression through TLR-TLR ligand interactions via a MyD88-dependent pathway in mice. This regulation can help decrease intestinal inflammation and maintain intestinal homeostasis. miR-10a targets IL-12/IL-23p40, a key molecule mediating innate immune responses [57].

Other studies have shown that the altered expression of some miRNAs such as miR-21, miR-122a, miR-155, and miR-150 in IBD and experimental colitis increases intestinal permeability by affecting tight junction proteins, leading to epithelial integrity loss, immune responses, and severe tissue damage. Additionally, the altered miR-126 and miR-146a expression further disrupts innate and adaptive immune responses in intestinal inflammation [58]. Thus, the gut microbiota has been reported to affect host health by modulating miRNAs. These can regulate the differentiation and function of various immune cells, making them crucial for both innate and adaptive immunity

Research related to the expression of miRNAs in different gastrointestinal tract processes in pigs has been carried out. Zhu et al. (2017) found that the expression of miR-29a increased in the jejunum of pigs with intrauterine growth restriction (IUGR), which also regulated this condition [56]. This miRNA regulates intestinal permeability in irritable bowel syndrome (IBS) patients [59]. Zou et al. (2019) determined that the expression of miR-100 is related to the regeneration of porcine intestinal epithelial cells; they found that miR-100 enhances differentiation and apoptosis while inhibiting the proliferation and migration of porcine enterocytes [60]. Tao et al. (2013) performed a transcriptomic analysis of the expression of miRNAs in the small intestine of lactating and weaned pigs. They found that miR-146b presented the most significant difference in expression between the two groups studied. This miRNA is related to functions such as regulating the inflammatory response, possibly by suppressing the expression of TLR4 [61]. In other words, when miR-146b increases, low levels of TLR4 are observed, which is related to the induction of proinflammatory responses. 

## 7. Modulation of miRNAs by Probiotics

Probiotic supplementation can modulate miRNA expression levels, contributing to maintaining intestinal microenvironment homeostasis. The impact of probiotics on specific miRNAs has been documented. Moreover, studies on probiotic supplementation effects on miRNAs are scarce, and as such, the results should be taken cautiously; some of them are summarized in Table 2.

For instance, Kreuzer-Redmer et al. (2016) investigated the differential expression of miRNAs and their target genes in the lymphatic tissue of the ileum and jejunum of piglets supplemented with the probiotic *E. faecium* NCIMB 10415. They found that miR-423-5p expression was upregulated and that the immunoglobulin lambda light chain (IGLC) and immunoglobulin kappa constant (IGKC) regions were downregulated in ileal lymph node cells. *IGLC* and *IGKC* are genes that encode the constant regions of immunoglobulins, which are crucial for B cell regulation. Furthermore, they identified IGLC as a target of miR-423-5p, a miRNA implicated in human cancer and heart diseases [62].

Rodríguez-Nogales et al. (2017) evaluated the effect of the probiotics *Limosilactobacillus fermentum* and *Ligilactobacillus salivarius* in mice with colitis induced by dextran sulfate sodium (DSS). Its administration improved the expression of certain miRNAs that were modified by inducing colitis. Both probiotics reduced the expression of miR-155 and miR-223. Only *L. fermentum* downregulated miR-150 and restored miR-143 expression [55]. Examples of the participation of these miRNAs, for instance, miR-155, fulfill different functions; among them, it is involved in regulating the immune response, controlling and developing innate immune cells, and responding to pro- and anti-inflammatory signals; its deregulation contributes to the appearance of different diseases such as chronic inflammation, cancer, and fibrosis. While miR-223 is positively regulated in inflammation, it is more related to the production of IL-1B [55,63]. Therefore, it is shown that probiotics can regulate specific miRNAs that are modified in intestinal pathogenesis.

Wang et al. (2020) identified a total of 12 miRNAs (miR-196b-5p, -196a, -20b, -9841-3p, -7857-3p, -206, -331-3p, -4334-3p, -144, -146b, let-7i-3p, and -21-3p) that were upregulated in the mucosa of the ileum in piglets upon administration of *L. reuteri*. Conversely, seven miRNAs (miR-10386, -1285, -490-5p, -218b, -338, -194a-3p, and -7-5p) exhibited negative regulation. Notably, miR-196a and miR-196b-5p demonstrated the highest levels, being four times more abundant than the other miRNAs [64]. This study also revealed that the target gene for these two miRNAs is the SOCS4 (Suppressor of Cytokine Signaling 4) protein. The SOCS family of proteins acts as negative regulators of proinflammatory cytokines. Therefore, the positive regulation of SOCS4 observed with *L. reuteri* administration suggests a potential modulatory mechanism in response to the probiotic [64].

Studies in pigs show that the probiotic *Bifidobacterium animalis* subsp. *lactis* (BB-12) regulates some miRNAs. For example, in BB-12-stimulated porcine monocyte-derived dendritic cells (moDCs), the miRNAs miR30b-3p and miR9858-5p were found to downregulate the expression of SLA-DR and CD80, two important molecules in antigen presentation. Additionally, miR671-5p targets IL-10 expression [65]. Even though miR-671-5p is a great candidate for regulating IL-10, the studies described were carried out in cells derived from peripheral blood. Therefore, the question arises as to which miRNAs could be responsible for producing IL-10 in the intestine, contributing to anti-inflammatory action and intestinal homeostasis.

**Table 2:** Changes in miRNA expression after probiotic supplementation.

Probiotic	miRNA Expression	Model	Reference
*Lactiplantibacillus plantarum* 299v	Increase in smiR-218b, miR-450a, miR-106a, miR-184, miR-9841-3p, miR-187. Decrease in miR-196a, miR-199a-3p, miR-218-5p, miR-194b-3p in ileum	Pig	[66]
*Saccharomyces boulardii*	Decrease in miR-155 y miR-223 in DSS-induced colitis	Mice	[58]
*L. fermentum* and *L. salivarius*	Decrease in miR-155 and miR-223 in DSS-induced colitis	Mice	[55]
*L. acidophilus* and *B. bifidum*	Decrease in miR-135b and miR-155, increase in miR-26b and miR-18a in AOM-induced colon cancer.	Mice	[67]
*E. faecium*	Increase in miR-149 and miR-1285 in jejunum. Increase in miR-423-5p and miR-1285 in lymph nodes.	Pig	[62]

DSS: dextran sulfate sodium; AOM: Azoxymethane.

In a study conducted by Arenas-Padilla et al. (2022), miR-15a-5p, miR-29b-3p, miR-30d-5p, and miR-181a-5p were found to be upregulated by BB-12 in porcine moDCs [68]. Notably, overexpression of miR-15a-5p acts as a tumor suppressor by inhibiting B-cell lymphoma 2 (Bcl-2), an anti-apoptotic protein whose expression can be stimulated by NF-κB [69,70,71]. Reduced levels of miR-29b-3p are associated with decreased cell proliferation and an increase in apoptosis and autophagy processes [72]. The positive expression of miR-30d and miR-181a regulates MAP4K4, MAPK1, and MAP2K1 (mitogen-activated protein kinases). These proteins belong to the MAP kinase family and can be activated by TLR2 [73]. Expression of pERK1/2 (extracellular signal-regulated kinase) proteins upon BB-12 stimulation in porcine moDCs may be involved in IL-10 production. So, it is suggested that the probiotic BB-12 can induce the expression of miRNAs that regulate proteins of the MAP kinase pathway. Furthermore, the production of IL-10 can be controlled by the activation of ERK1/2. Furthermore, when TLR2 is blocked, the expression of pERK1/2 decreases; this protein is activated by stimulating this receptor [68].

The MAP kinase family of proteins plays an important role in the activation of the transcription factors NF-κB and AP1. They are involved in TLR2 signaling and cytokine production. It is suggested that BB-12 modulates some proteins of the MAP kinase pathway, such as MAPK1 and pERK1/2, which are important to produce IL-10 and can be regulated by miR-30d and miR-181. The latter and miR-155 are expressed in pig tissues such as the spleen, muscle, peripheral blood mononuclear cells, and lymph nodes [74]. BB-12 immunomodulation follows a pathway that miRNAs can regulate and drive an anti-inflammatory response. The suggested pathway is recognition by TLR2 and activation of the adapter molecules MyD88 and TRAF6. This is followed by the activation of pERK1/2, which activates AP1, and finally, the production of IL-10 with the possible regulation by miR-30d and miR-181. Therefore, it is suggested that the MAP kinase pathway could participate in regulating homeostasis in the intestine.

Thus, probiotics play a pivotal role in modulating the expression of miRNAs within intestinal cells, which in turn regulate various biological processes essential for maintaining gut homeostasis. By influencing miRNA expression, probiotics can exert significant anti-inflammatory effects, enhance immune responses, and contribute to the host’s overall health. This modulation occurs through intricate signaling pathways that adjust gene expression at the post-transcriptional level. Understanding these mechanisms offers insights into the therapeutic potential of probiotics in managing gastrointestinal disorders and other related health conditions.

## 8. Perspectives and Conclusions

The use of probiotics as supplements has increased over the past decades in human and animal feeding. In animals used for human consumption, such as pigs, most documented effects deal with weight gain and overall health increase, but few studies explore the mechanism of action. In this review, we have focused on the physiological aspects of intestinal homeostasis and presented some of the few studies on the use of probiotics in pigs focusing on immune response. Exploring the interactions between microbiota–probiotics–ncRNA/protein opens the panorama to understand the regulatory mechanisms of probiotics in the host. Analyzing small RNAs, such as miRNAs, as important regulators leads to post-transcriptional changes that affect the expression of proteins involved in cell signaling pathways. These pathways can, in turn, regulate the expression of other proteins or molecular mediators. Probiotics can regulate the expression of specific proteins by modulating miRNAs or other ncRNAs, which may act as a feedback mechanism, partly explaining their action at a deeper molecular level. For example, probiotics can alter the gut microbiota composition, influencing the host’s miRNA expression profiles. These miRNAs then target specific mRNAs, leading to their degradation or translational repression, thus finely tuning the protein output. This modulation affects various biological processes, including inflammation, immune response, and cellular homeostasis.

Moreover, certain probiotics may enhance the production of anti-inflammatory cytokines through miRNA-mediated pathways, contributing to intestinal homeostasis and reducing the risk of diseases such as IBD and colorectal cancer. The intricate balance maintained by these regulatory mechanisms highlights the potential therapeutic applications of probiotics in modulating gene expression and maintaining gut health. In summary, probiotics exert their beneficial effects through complex regulatory networks involving miRNAs and other ncRNAs, which orchestrate a cascade of molecular events crucial for maintaining intestinal health and overall homeostasis.

## Data Availability

Data sharing not applicable.
